# Time series modeling of pertussis incidence in China from 2004 to 2018 with a novel wavelet based SARIMA-NAR hybrid model

**DOI:** 10.1371/journal.pone.0208404

**Published:** 2018-12-26

**Authors:** Yongbin Wang, Chunjie Xu, Zhende Wang, Shengkui Zhang, Ying Zhu, Juxiang Yuan

**Affiliations:** 1 School of Public Health, North China University of Science and Technology, Tangshan, Hebei Province, P.R. China; 2 School of Public Health, Capital Medical University, Beijing, P.R. China; University of Rochester, UNITED STATES

## Abstract

**Background:**

It is a daunting task to discontinue pertussis completely in China owing to its growing increase in the incidence. While basic to any formulation of prevention and control measures is early response for future epidemic trends. Discrete wavelet transform(DWT) has been emerged as a powerful tool in decomposing time series into different constituents, which facilitates better improvement in prediction accuracy. Thus we aim to integrate modeling approaches as a decision-making supportive tool for formulating health resources.

**Methods:**

We constructed a novel hybrid method based on the pertussis morbidity cases from January 2004 to May 2018 in China, where the approximations and details decomposed by DWT were forecasted by a seasonal autoregressive integrated moving average (SARIMA) and nonlinear autoregressive network (NAR), respectively. Then, the obtained values were aggregated as the final results predicted by the combined model. Finally, the performance was compared with the SARIMA, NAR and traditional SARIMA-NAR techniques.

**Results:**

The hybrid technique at level 2 of db2 wavelet including a SARIMA(0,1,3)(1,0,0)_12_modelfor the approximation-forecasting and NAR model with 12 hidden units and 4 delays for the detail d1-forecasting, along with another NAR model with 11 hidden units and 5 delays for the detail d2-forecasting notably outperformed other wavelets, SARIMA, NAR and traditional SARIMA-NAR techniques in terms of the mean square error, root mean square error, mean absolute error and mean absolute percentage error. Descriptive statistics exhibited that a substantial rise was observed in the notifications from 2013 to 2018, and there was an apparent seasonality with summer peak. Moreover, the trend was projected to continue upwards in the near future.

**Conclusions:**

This hybrid approach has an outstanding ability to improve the prediction accuracy relative to the others, which can be of great help in the prevention of pertussis. Besides, under current trend of pertussis morbidity, it is required to urgently address strategically within the proper policy adopted.

## Introduction

Pertussis, or whooping cough, is an acute infectious disease of the respiratory tract caused by *Bordetella pertussis*[1]. This disease can easily achieve transmission from child to child by means of the droplets fueled by coughing or sneezing of the infected[1]. Before pertussis was widely vaccinated, it is among the most dangerous diseases with high morbidity and mortality in infants and young children due to such severe complications as pneumonia, encephalopathy, apnea, and pulmonary hypertension[2, 3]. The available vaccine was introduced to this preventable disease since 1953, aforementioned worsening situation has well been controlled[3]. At present, albeit the well-established vaccine coverage has reached 86% on a global scale in 2016, a large proportion of childhood death tolls can still be directly or indirectly attributed to pertussis[4, 5]. Globally in 2017, there are 131991 notified cases with 89000 estimated deaths linked to the disease, and about 95% of them were notified in developing countries[4]. Furthermore, the past decade has witnessed a growing tendency for the incidence of pertussis in many places of the world, such as America, France, Germany, India, Canada, China and Australia and so on[6, 7]. Of these countries, outbreaks are frequently observed as well with a circle of every 2 to 5 years[8]. As such resurgence of pertussis incidence, this disease has caused wide public concern and is still viewed as a major public health issue in the world. China is well received as a fast-developing country with the largest-scale children worldwide[9], several reports have indicated that the morbidity rate of pertussis has continued to increase over the recent years[5, 7]. Importantly, according to the World Health Organization(WHO) monitoring data in 2017, among 194 countries standing the fourth rank in pertussis infections is China, with an estimated 10390 cases, or so amounting to approximately 8% of the total[4]. Furthermore, epidemiological investigations have showed that the incidence of pertussis in China is seriously underestimated to a great extent[10–12]. Facing with the impact imposed by pertussis in increasing numbers on the Chinese children, there is an imminent need to tackle such a wide-ranging threat. While advanced warning is invariably deemed as a significant step towards the prevention and control of diseases. Therefore, it is under the imperative to develop a computational model with robust accuracy and precision to analyze and detect its epidemic patterns of pertussis in the coming years, which will play a pivotal role in minimizing the potential health hazards and economic losses induced by the disease.

Currently, plenty of mathematical techniques have already been considered as a tool of growing interest in the domain of epidemiological forecasting. Among which the classical seasonal autoregressive integrated moving average (SARIMA) method that is one of the best-known essentially linear models has extensively been employed to predict the incidence of contagious diseases[13]. Generally, the time series data are often characterized by the time-varying aspects of secular trend, cyclicity, seasonality and stochastic fluctuation, which make the data be made up of the linear and nonlinear traits[5]. Whereas the SARIMA model assuming time series to be stationary is not adept at excavating the elements of non-stationarity and non-linearity hidden behind this incidence data[13]. Consequently, to obtain a satisfactory estimation result, it is important to heighten the need for the extraction of the non-linear relationship in this time series. While owing to the flexible capability of artificial neural networks (ANNs) to unearth the non-stationary and non-linear implications of a time series, they thus are quite appropriate to deal with such type of nonlinear relation in time series [14]. Nowadays, among the ANNs methods revealing outstanding simulation and prediction accuracies in the field of epidemiology is the nonlinear autoregressive network (NAR) technique[15]. However, when a mathematical methodology was applied to understand the future temporal levels of infectious diseases, neither the linear nor nonlinear techniques seemingly fail to be satisfactory[13]. Therefore, to enhance the prediction performance, the preferred estimation tool is supposed to be tailored for those various time series elements[16]. More recently, there has been an increasing interest in the potential applications of a discrete wavelet transform (DWT)for filtering and preliminary handling of raw data to extract meaning clues particularly when time series shows the non-stationary and non-linear, as DWT can attain to a capacity that discerns exceptional events (outliers) by time-localized frequency analysis[17–19]. Thereby, inspired by the merits of this technique and in light of the traits of pertussis incidence, we aimed to set up a novel DWT based SARIMA-NAR hybrid model to forecast the epidemic characteristics of pertussis morbidity, i.e., the DWT approaches were used to classify the reported pertussis series into the high-scale and low-frequency constituents that stand for the approximate scale of the signal(linear part), along with the low-scale and high-frequency constituents that refer to the detailed scale of the signal(nonlinear part)[16]. Whereafter, the informative clues of the approximations were extracted utilizing a SARIMA model, whereas the useful clues of the details were excavated with a NAR model. Ultimately, the forecasted results of the basic SARIMA and NAR models were aggregated as the predictive values for the actual pertussis time series. By doing so, this data-driven combined model could comprehensively take advantage of the linear and nonlinear information in the pertussis time series. Whilst in order to compare the excellent performance of our proposed combined approach in pertussis modeling, the basic SARIMA and NAR approaches, as well as the traditional SARIMA-NAR hybrid technique were also tested for their capability to effectively forecast pertussis morbidity.

## Materials and methods

### Data collection

In this research, the monthly and yearly pertussis cases aggregated at national level from January 2004 to May 2018 were obtained from the notifiable infectious disease reporting system(http://www.nhfpc.gov.cn/jkj/s3578/new_list.shtml**)**, and annual population data during the period 2004–2017 were retrieved from National Bureau of Statistics of China (http://data.stats.gov.cn/easyquery.htm?cn=C01).A total of 173 reported observations spanning 15 years were summarized and collated (**S1 Table**). Subsequently, to test the fitting and forecasting performances among these four models, the longitudinal notified data involving 167 months were employed to recognize the optimal parameters of models, while the remaining 6 data points were used to examine the models’ generalization of ability. In China, all cases confirmed through the clinical or laboratory diagnostic criteria for pertussis must be notified online within 24 hours, and then must be checked by the professionals from local Center for Disease Control and Prevention (CDC). The ethical approval or consent is not needed for our present study as the monthly or annual reported cases of pertussis are publicly available in China.

### Building SARIMA model

The SARIMA model-constructing procedure is designed to consider the associations in the sequentially lagged relationships that often exist in cyclically aggregated time series[20]. Since the morbidity time series of infectious diseases commonly includes significant cyclical and seasonal patterns, the basic SARIMA method should be considered. The basic form of a SARIMA method used for modeling pertussis morbidity cases can be defined as SARIMA(p, d, q) (P, D, Q)s[21]. Presented herein are the length of periodicity (S), non-seasonal and seasonal differenced times(d and D, respectively), non-seasonal autoregressive model(AR) and moving average model(MA) (p and q, respectively), coupled with the orders(P and Q, respectively) of seasonal autoregressive model(SAR) and moving average model(SMA). In our work, SPSS software (version 17.0, IBM Corp, Armonk, NY) was adopted to erect the SARIMA model as it allows us to adopt a straightforward solution to identifying the significant parameters. Firstly, after performing the “Expert Modeler” function in SPSS software, it would opt for the best-fitting SARIMA among all the alternative models based on the goodness of fit tests with the largest value of R-squared(R^2^) and the lowest value of normalized Bayesian information criterion(BIC),and the suitable autocorrelation function(ACF) and partial autocorrelation function(PACF) plots of the residuals[21]. Then, the Ljung-Box *Q*-test was employed to validate whether the estimated residuals satisfied the requirement of a white-noise sequence. Finally, the chosen SARIMA could be considered as the best-performing to compute the out-of sample forecasts. The equation of a SARIMA(p,d, q) (P, D, Q)_s_ model can be of the form[22]

$$\begin{array}{l} \phi \left(B\right)\Phi \left(B^{s}\right)\Delta^{d}\Delta_{s}^{D}X_{t}=\theta \left(B\right)\Theta \left(B^{s}\right)\varepsilon_{t}\\ E\left(\varepsilon_{t}\right)=0,\;Var\left(\varepsilon_{t}\right)=\sigma_{\varepsilon }^{2},E\left(\varepsilon_{t}\varepsilon_{s}\right)=0,s\neq t\\ E\left(X_{s}\varepsilon_{t}\right)=0,\forall_{s}<t \end{array}$$ (1)

Where B signifies the backward shift operator, ɛ_t_ denotes the residual errors from the SARIMA method, S, d, D, p, q, P and Q are what as illustrated above, respectively.

$$\nabla^{d}={\left(1-B\right)}^{d},\;\nabla_{S}^{D}={\left(1-B\right)}^{SD},\;\phi \left(B\right)=1-\phi_{1}B-\cdots -\phi_{p}B^{p},\;\theta \left(B\right)=1-\theta_{1}B-\cdots -\theta_{q}B^{q},\;\Phi \left(B^{s}\right)=1-\Phi_{1}B^{s}-\cdots -\Phi_{P}B^{Ps},\;\Theta \left(B^{s}\right)=1-\Theta_{1}B^{s}-\cdots -\Theta_{Q}B^{Qs}.$$

### Establishing NAR model

ANNs models have attracted much attention in the field of forecasting due to their powerful nonlinear mapping ability and have been regarded as a nonlinear regression technique capable of approximating arbitrarily intricate non-stationary series to attain any desired accuracy[23]. Of all the ANNs methods, the NAR model belonging to one of the foremost dynamic neural networks with a tapped delay line represents a leading class of methods that can accomplish arbitrary nonlinear dynamical mappings and be fairly skilled in estimating dynamical system[24]. In this network, it uses tapped delay lines to store previous values of the y(t) sequences, and the predictive outputs are fed back into the network as inputs at the next time step. When the network is based separately on the current state of the variable for which a forecast is made, the external inputs are only the current state of the variable. During use of this technique, the network recursively uses its own outputs as inputs for the length of time for which a forecast is necessary. The specified equation of a NAR model can be defined as:

y(t)=f(y(t−1),y(t−2),⋯,y(t−d)) (2)

Where, y(t) represents the predicted points of pertussis incidence series depended merely on the prior data of lagged period *d*.

When employing a NAR approach, the in-sample observations were broken down into training, validation and testing datasets with the ratio of 80%, 10% and 10% respectively to model the optimum parameters in an open-loop mode. Of these datasets, the training subset was employed to determine the network parameters; the validation subset was applied to improve the model’s generalization by avoiding overfitting; the testing subset provided an independent measure of the model performance. Next, the training open-loop form should be closed to undertake multi-step-ahead estimations[24]. The closed-loop equation of a NAR model can be specified as:

y(t+p)=f(y(t−1),y(t−2),⋯,y(t−d)) (3)

Here, *p* is the out-of-sample outputs.

### Constructing traditional SARIMA-NAR combined model

As depicted above, the SARIMA model commonly does well in extracting the linear clues hidden behind the pertussis morbidity time series[13].Consequently, the nonlinear information is included in the residuals-generating series, which may make the SARIMA fail to effectively analyze this component under study[25]. Fortunately, the NAR method viewed as a powerful dynamic function approximator can effectively extract this component[23]. In order to overcome the limitation of a basic SARIMA method used solely, a hybrid SARIMA-NAR approach is thus developed to comprehensively take advantage of the unique strength of the single SARIMA and NAR methods during the process of modeling pertussis morbidity time series. In modeling this hybrid approach, since there are either positive or negative numbers in the residual error series of the SARIMA model, which increases the difficulty of training this model. Hence to efficiently model this data, we adopted the normalized method[14] to preprocess the data into [0, 1] intervals. Subsequently, the preprocessed residual error series was applied to construct the single NAR method[23], and the modeling procedure and model performance comparison were the same as the basic NAR technique. Then, the obtained results were implemented with the reverse normalization method. Finally, the values fitted and predicted by the single SARIMA and NAR methods respectively were summed to be referred to as the final pertussis morbidity cases derided from the SARIMA-NAR model. This combined SARIMA-NAR method can be expressed as:

$$\hat{e}(t)=f(e(t-1),e(t-2),\cdots ,e(t-d))$$ (4)

$$\hat{y}={\hat{a}}_{t}+{\hat{e}}_{t}$$ (5)

Where $\hat{y}$, ${\hat{a}}_{t}$ and ${\hat{e}}_{t}$ refer to the mimic and estimated results using the combined SARIMA-NAR, single SARIMA and NAR methods, respectively.

### Wavelet based SARIMA-NAR hybrid model formulation

Recent developments in data-processing have led to a renewed interest in wavelet transform, which can act as a beneficial approach for signal analysis in both time and frequency[19]. The theory of this technique is formed relied on Fourier analysis[17]. While the method can provide the local clues of a signal at various scales and show an excellent flexibility in choosing a mother wavelet based on the properties of a time series as compared to the Fourier analysis[18]. Because of such desirable traits of a wavelet, it is especially appropriate to handle any sort and size of time series[19]. Generally, wavelet analysis manifests favorable performance in exploring, de-noising and smoothening time series, thus better displaying the trends, break-down points and uncontinuity within the time series that other types of signal analysis technique might leave out and having played a paramount role in prediction and other empirical analysis[19]. Wavelet transformation approaches are chiefly composed of continuous wavelet transformation (CWT) and DWT[18]. The wavelet transform coefficients calculated by CWT often contain a lot of redundant information, which leads to a positive correlation among these coefficients and is detrimental to feature compression and numerical calculation[18]. Whereas the scale and displacement parameters can be discretized and then wavelet transformed in the process of DWT, which can reduce the redundancy of the wavelet transform coefficients[17]. Thereby, in practice, more attention has been poured in the DWT.

In this project, our proposed wavelet based SARIMA-NAR hybrid model was established with MATLAB software(Version R2014a, MathWorks, Natick, MA, USA): Initially, the DWT method was employed to decompose the reported pertussis incidence series into one comprising its secular changes(the approximate component) and the other comprising the high frequencies and the fast events(the detailed component). Secondly, the SARIMA model was used to mine the linear trend information in the approximate subseries. While the NAR method was applied to extract the remaining non-linear detailed implications in the detailed subseries. Finally, the estimation results emerged from the basic SARIMA and NAR models were summed to become the forecasting values for the original pertussis time series.

### Assessing models performance

The mimic and predictive effects of these selected methods were measured with the absolute error (AE), mean square error (MSE),root mean square error (RMSE), mean absolute error (MAE) and mean absolute percentage error (MAPE). The lower the indices’ values are, the better the approaches are.

$$\mathrm{AE}=\sum_{i=1}^{N}\left|X_{i}-{\bar{X}}_{i}\right|$$ (6)

$$\mathrm{MSE}=\frac{1}{N}\overset{N}{\underset{i=1}{\sum }}{\left(X_{i}-{\bar{X}}_{i}\right)}^{2}$$ (7)

$$\mathrm{RMSE}=\sqrt{\frac{1}{N}\overset{N}{\underset{i=1}{\sum }}{\left(X_{i}-{\bar{X}}_{i}\right)}^{2}}$$ (8)

$$\mathrm{MAE}=\frac{1}{N}\sum_{i=1}^{N}\left|X_{i}-{\bar{X}}_{i}\right|$$ (9)

$$\mathrm{MAPE}=\frac{1}{N}\sum_{i=1}^{N}\frac{|X_{i}-{\bar{X}}_{i}}{X_{i}}$$ (10)

Here, *X*_*i*_ is the actual case notifications, ${\bar{X}}_{i}$ denotes the fitted and forecasted morbidity cases with the chosen models, *N* refers to the number of mimics and predictions.

## Results

### General information

**Fig 1:**
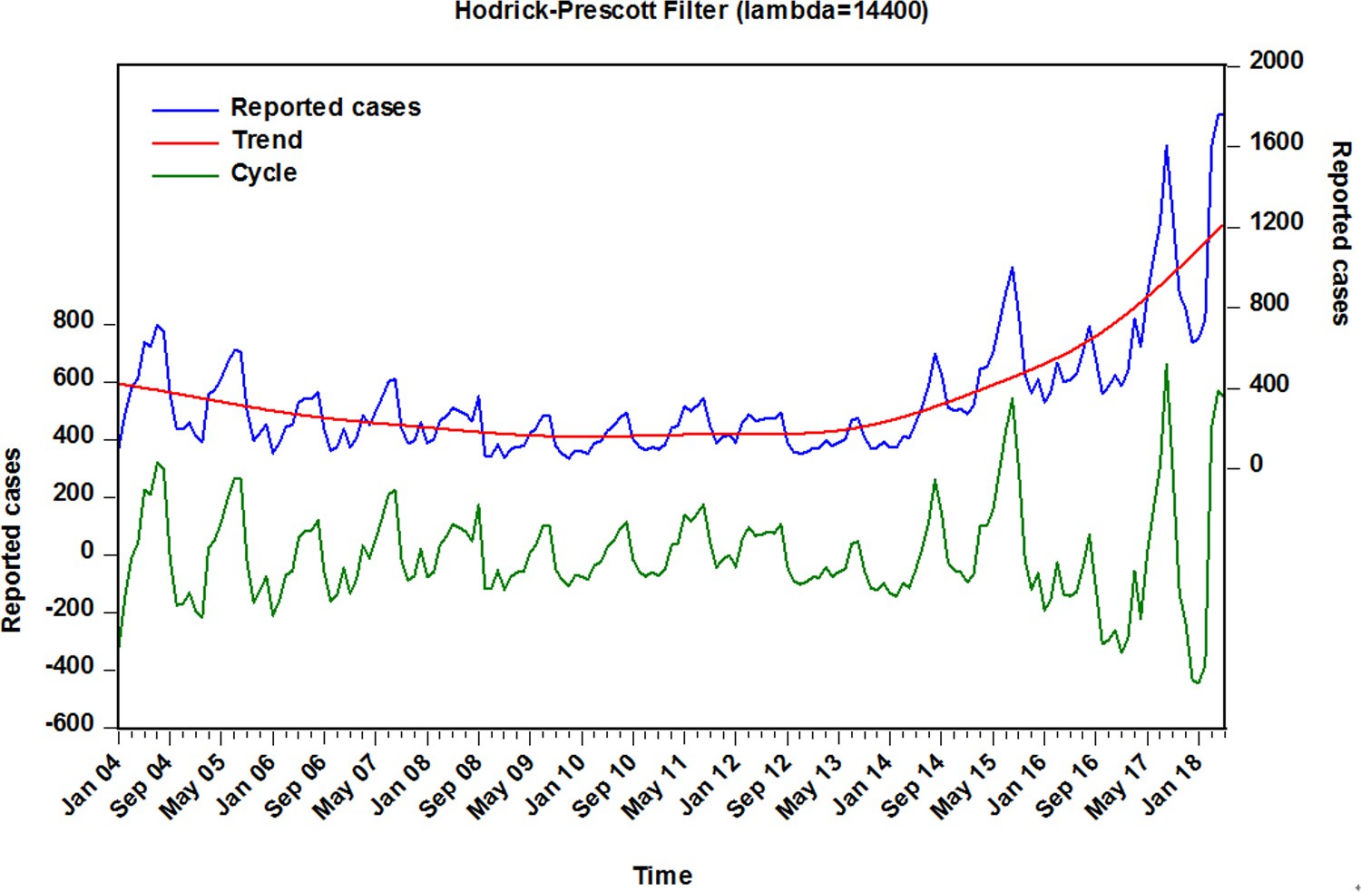
The notified monthly cases of pertussis and decomposed long-term trend and cyclicity with Hodrick-Prescott filter methodology from January 2004 to May 2018 in China. Blue line represents the reported cases; red line stands for the decomposed long-term trend of the notified monthly cases of pertussis, it can be seen that a remarkably upward trend was observed from 2013 to 2017; green line is the decomposed cyclical process with a length of 12 months.

Between January 2004 and May 2018, a totalof60178 pertussis cases were notified with an average monthly reported cases of 348(average annual incidence rate was 0.277 per 100000 population). According to the monthly pertussis cases notified in China, the number of cases increased considerably from 4075 in 2004 to 10390 in 2017with a rise by 120.829%. When the Hodrick-Prescott method was employed to remove the short-term monthly effect of pertussis morbidity, indicating that a slightly downward trend was seen from 2004 to 2013, while a remarkably upward trend was reported from 2013 to 2017with 506.893% upsurge than that of 2013 when it was only 1712 in all(**Fig 1**). Additionally, a trough was commonly observed in January, February, October, November and December per year(autumn and winter), especially in January with the lowest level. Whereas the peak activities frequently existed from March to September per year(spring and summer), particularly in August with the climax(**S1 Fig**).

### The best-performing SARIMA model

**Fig 2:**
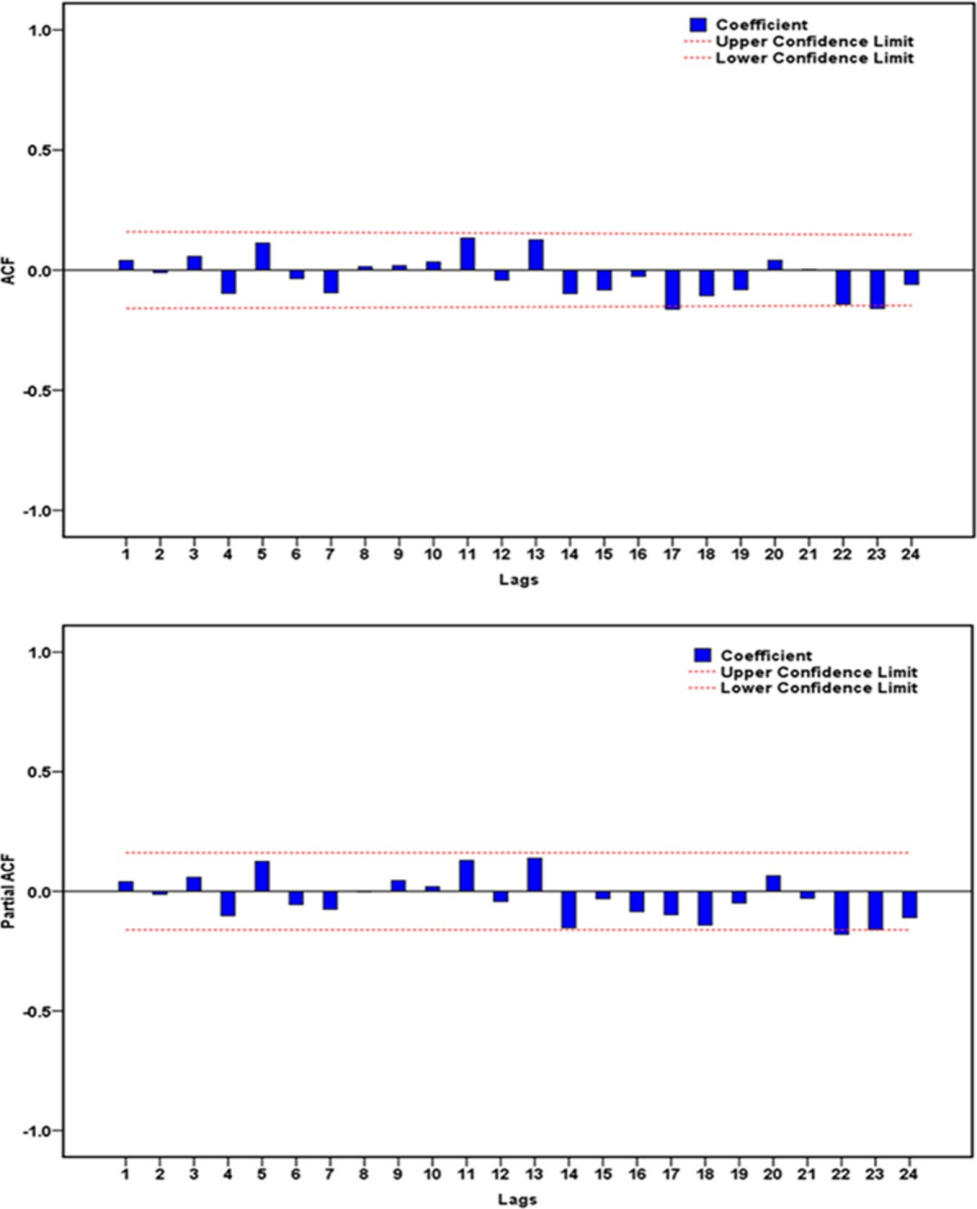
The resulting autocorrelation function (ACF) and partial autocorrelation function (PACF) graphs of residuals from SARIMA(2,1,0)(0,1,1)_12_ model for the observatory pertussis morbidity time series. Almost all spikes fell within the estimated 95% uncertainty bounds at varying lags apart from the correlations at 17 and 23 lags, exhibiting that there appeared to be no correlated residuals. With respect to the spikes at 17 and 23 lags, it is also reasonable because two higher-order correlation functions may occasionally exceed the estimated confidence intervals.

By running the “Expert Modeler” functionin SPSS software, the optimal model was automatically identified as SARIMA(2,1,0)(0,1,1)_12_with the largest R^2^of 0.940 and the lowest normalized BIC of 8.348. The results to emerge from the ACF and PACF plots of residuals exhibited that there appeared to be no correlated residuals(**Fig 2**). The Ljung-Box *Q*-tests further suggested that the residual error series completed a white noise(**Table 1**). Moreover, as displayed in **Table 2,** which showed that the modeling parameters were all statistical significance. These results verified that the chosen best-performing SARIMA(2,1,0)(0,1,1)_12_ model was well suited to forecast the future temporal patterns of pertussis. The equation of this SARIMA approach was expressed as (1−*B*)(1−*B*^12^)*X*_*t*_ = (1−0.678*B*^12^)*ε*_*t*_/(1+0.448*B*+0.201*B*^2^).

**Table 1:** Ljung-Box *Q* tests of the residuals for the elected four models.

Lags	SARIMA		NAR		Traditional SARIMA-NAR		Wavelet based SARIMA-NAR	
	Box-Ljung *Q*	*P*	Box-Ljung *Q*	*P*	Box-Ljung *Q*	*P*	Box-Ljung *Q*	*P*
1	0.249	0.618	1.809	0.179	1.095	0.295	0.025	0.874
3	0.787	0.853	2.409	0.492	1.180	0.758	1.047	0.790
6	4.499	0.610	5.870	0.438	7.532	0.274	5.561	0.474
9	6.051	0.735	7.010	0.636	10.359	0.322	10.062	0.345
12	9.532	0.657	8.343	0.758	11.176	0.514	18.761	0.094
15	15.066	0.447	9.869	0.828	14.973	0.453	19.779	0.181
18	21.814	0.240	11.815	0.857	19.217	0.379	25.382	0.115
21	23.275	0.330	18.371	0.625	19.950	0.524	28.123	0.137
24	32.254	0.121	31.425	0.142	24.973	0.407	28.979	0.221
27	33.317	0.187	31.847	0.238	26.080	0.514	29.594	0.333
30	33.556	0.299	36.061	0.206	27.704	0.586	31.058	0.413
33	35.564	0.348	37.148	0.284	28.561	0.688	33.566	0.440
36	42.281	0.218	47.161	0.101	34.031	0.563	34.576	0.536

SARIMA, seasonal autoregressive integrated moving average model; NAR, nonlinear auto-regressive neural network model; Wavelet based SARIMA-NAR, integrating a seasonal autoregressive integrated moving model with a nonlinear autoregressive network model at level 2 of db2 wavelet.

**Table 2:** Estimated parameters of the SARIMA(2,1,0)(0,1,1)_12_ model.

Parameters	Coefficient	Standard error	*t*	*P*
AR1	-0.448	0.079	-5.662	<0.001
AR2	-0.201	0.079	-2.551	0.012
SMA1	0.678	0.072	9.427	<0.001

AR1, moving average, lag1; AR2, moving average, lag2; SMA1, seasonal moving average, lag12.

### The best-performing basic NAR model

**Fig 3:**
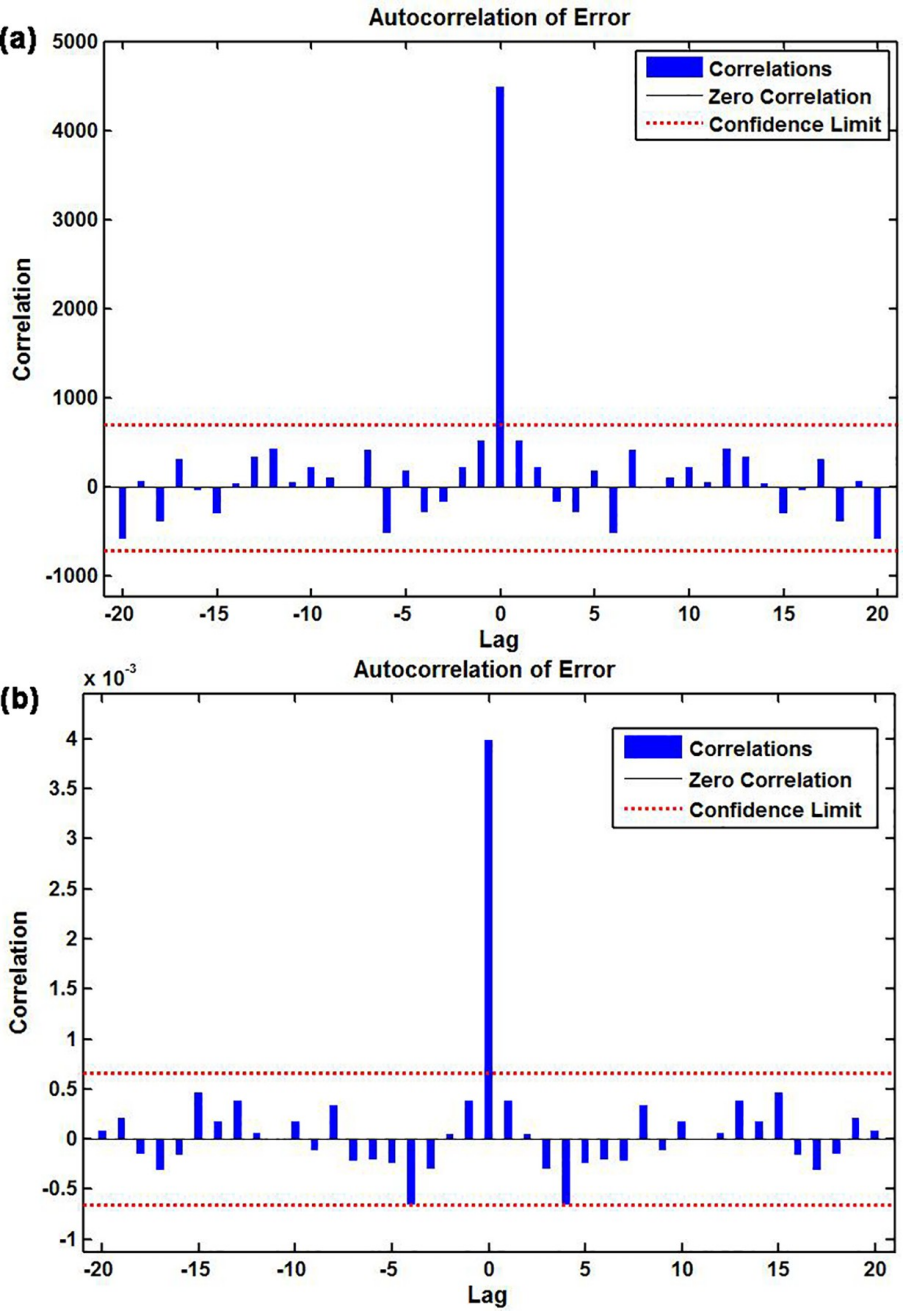
Autocorrelation function (ACF) graph of residuals for the observatory pertussis morbidity time series. (a) ACF graph of residuals from the basic NAR model; (b) ACF graph of residuals from the traditional SARIMA-NAR hybrid method.Theobservatory spikes aside from the one at zero lag failed to exceed the estimated 95% uncertainty intervals, so these two networks appear to be suitable.

**Fig 4:**
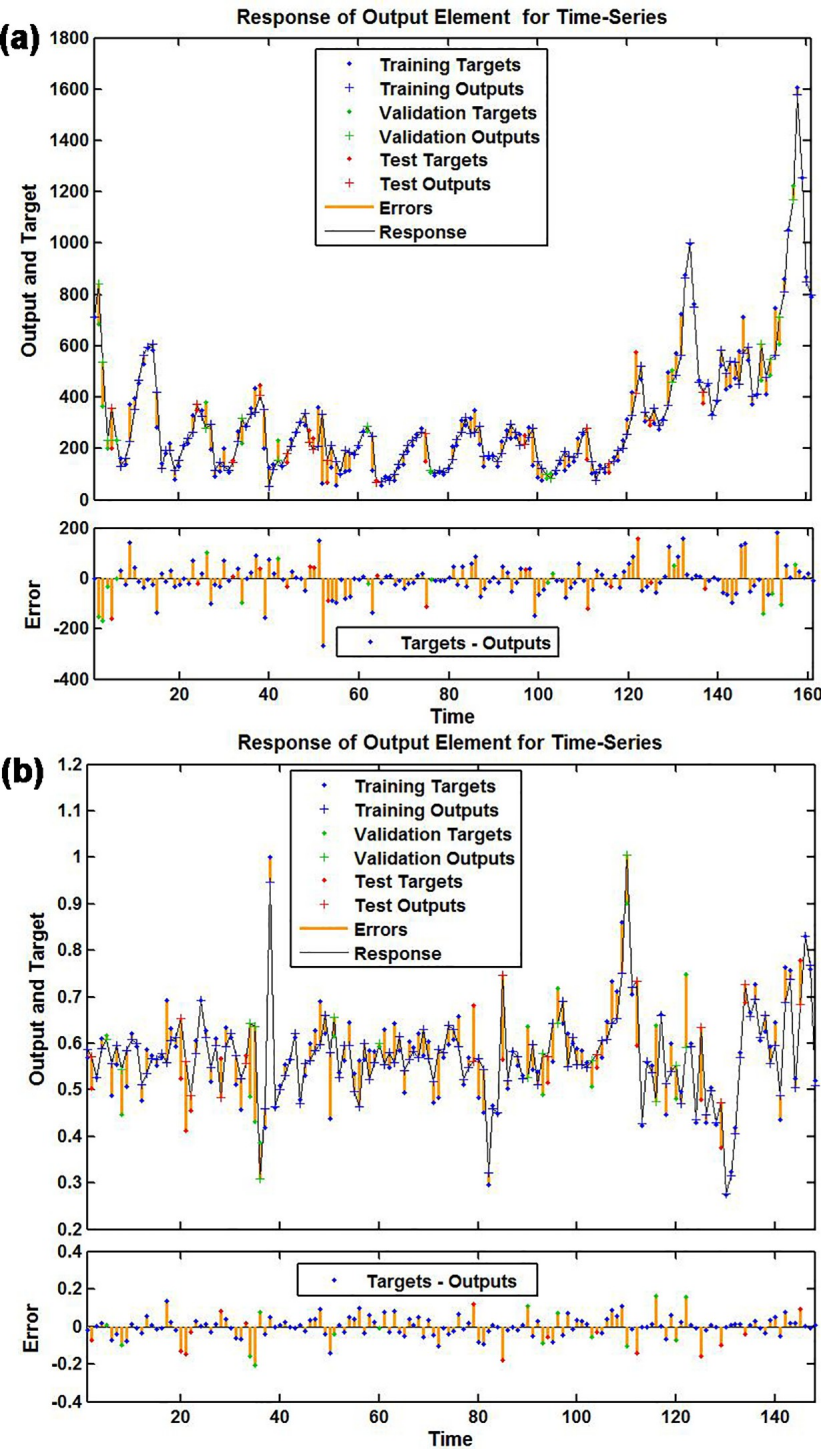
Response results of outputs and targets for the observatory pertussis morbidity time series. (a) Response graph from the basic NAR model; (b) Response graph from the traditional SARIMA-NAR hybrid method. The plots exhibited which time points were chosen as the training, validation and testing datasets, coupled with their corresponding errors between inputs and targets. In view of the small errors suggesting the prediction is accurate.

To discover a best-fitting NAR network, the ranges of the hidden units and feedback delays were set to 10–40 and 2–12, respectively. After training with the in-sample observations repeatedly, we found that the model with 16 hidden neurons and 6 feedback delays should be identified as the preferred basic NAR network, following the chosen optimal model were the minimum MSE values of training for 3930.093, validation for 7460.696, testing for 5977.639 and all data points for 4484.444, along with the maximum R values of training, validation, testing and entire datasets of 0.969, 0.962, 0.834 and 0.964, respectively (**S2 Fig**). The ACF plot of residuals reveals that the remaining autocorrelations were not observed because all spikes failed to exceed the estimated uncertainty intervals(**Fig 3A**). The Ljung-Box *Q*-statistics demonstrated that the residual series achieved a white noise (**Table 1**). Besides, the response results of outputs and targets manifested that overall the epidemic trends of pertussis morbidity were well tracked by the selected technique because of the small errors generated(**Fig 4A**). All resulting results suggested that a suitable NAR model was chosen.

### The best-performing traditional SARIMA-NAR hybrid method

According to the modeling procedure, by repeatedly training the feedback network, the best-simulating SARIMA-NAR technique with 23 hidden neurons and 6 feedback delays followed by the lowest training score for MSE = 0.002, validation for MSE = 0.012, testing for MSE = 0.011 and entire data for MSE = 0.004, along with the maximum R values of training, validation, testing datasets and all data of 0.898, 0.682, 0.543, and 0.813, respectively was chosen(**S3 Fig**).Goodness of fit tests for this combined method: the ACF graph demonstrates that all spikes at different lags, aside from the zero lag where it should occur, were within the 95% interval forecasts, suggesting that the model appeared to be adequate in modeling the data(**Fig 3B**). The Ljung–Box *Q*-statistics with all *P-*values of more than 0.05 showed that the model did not suffer from any correlated residuals(**Table 1**), a further indication that the model has captured the dependence in the time series. The response plot of the output elements for the randomly chosen training, validation and testing subsets exhibited the overall epidemic behaviors of pertussis incidence were well captured by this model owing to the minor residual errors lying between -0.2 and 0.2(**Fig 4B**). These diagnostics indicated that an adequate model was selected.

### The best-performing wavelet based SARIMA-NAR hybrid method

**Fig 5:**
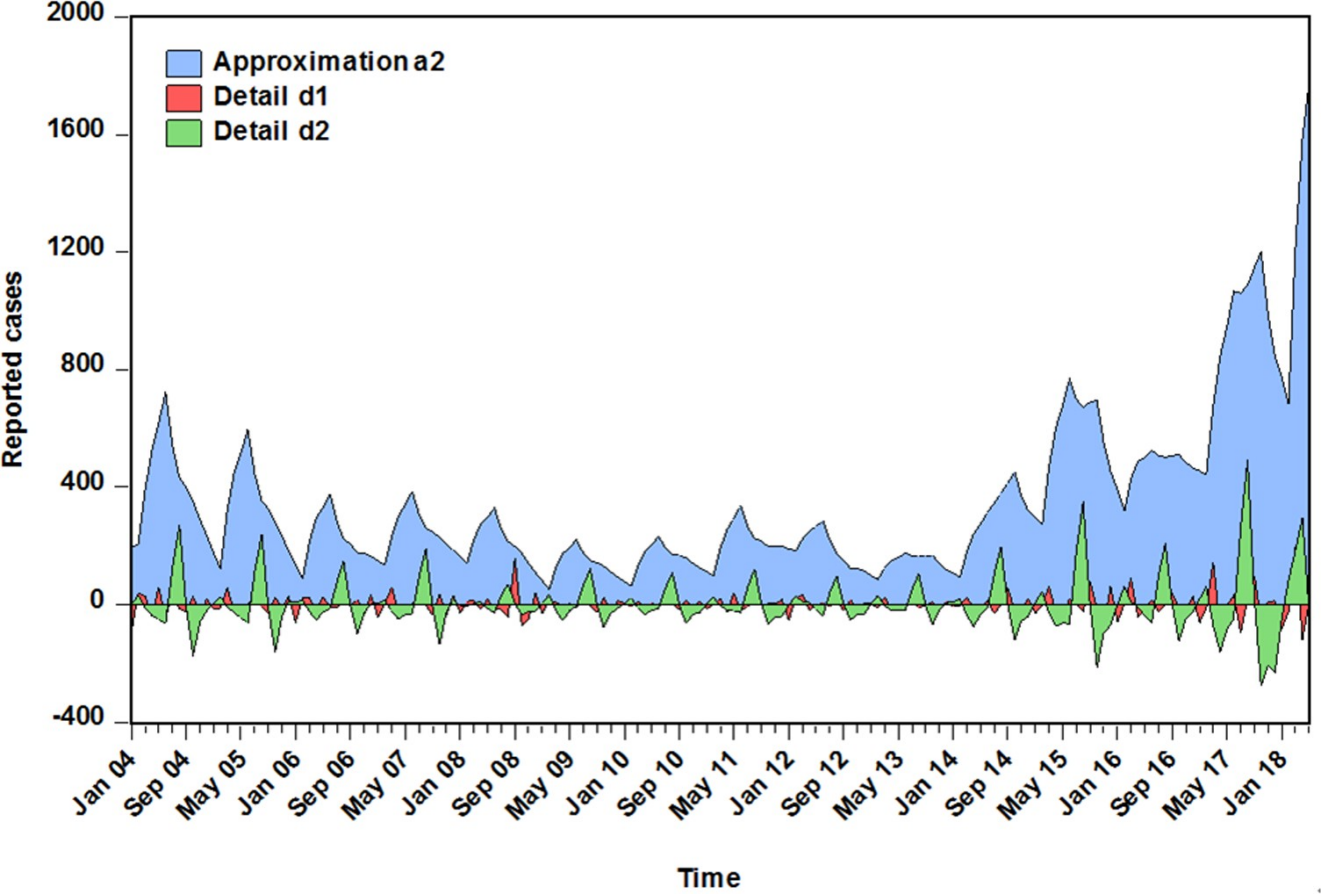
The decompositions at level 2 of db2 wavelet for the observatory pertussis morbidity cases time series. This plot displayed that the approximate element a2 stored the entire form of the pertussis morbidity cases time series, while the detailed components d1 and d2 showed the noise information of the pertussis reported series.

**Fig 6:**
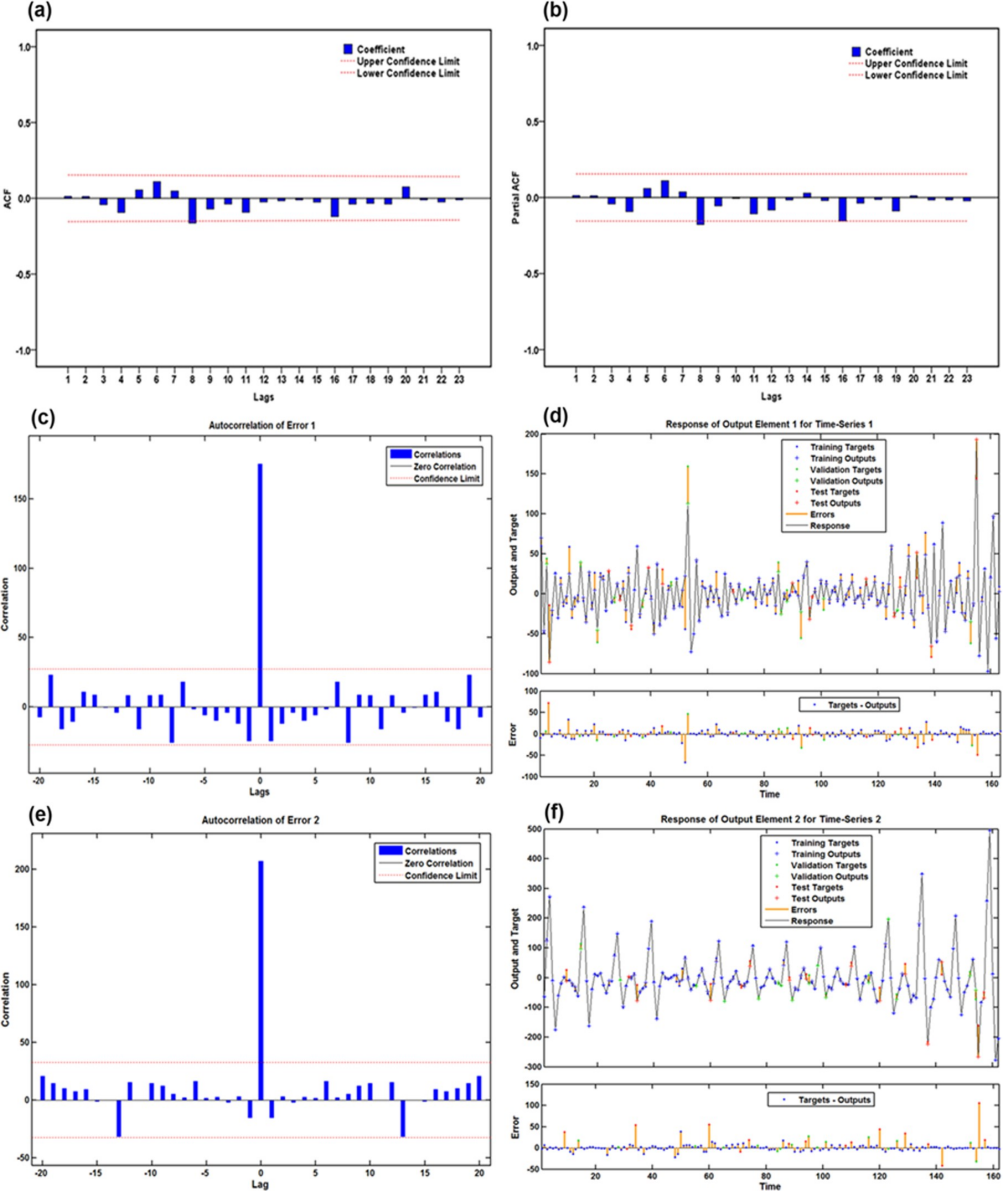
**Diagnostic tests of the corresponding individual models for the approximation and details yielded by db2 wavelet:** (a) Autocorrelation function (ACF) graph of residuals from the SARIMA(0,1,3)(1,0,0)_12_ model for the approximation; (b) Partial autocorrelation function (PACF) graph of residuals from the SARIMA(0,1,3)(1,0,0)_12_ model for the approximation; (c) Autocorrelation function (ACF) graph of residuals from the NAR model for the detailed component d1; (d) The response results of outputs and targets from the NAR model for the detailed component d1; (e) Autocorrelation function (ACF) graph of residuals from the NAR model for the detailed component d2; (f) The response results of outputs and targets from the NAR model for the detailed component d2.

**Fig 7:**
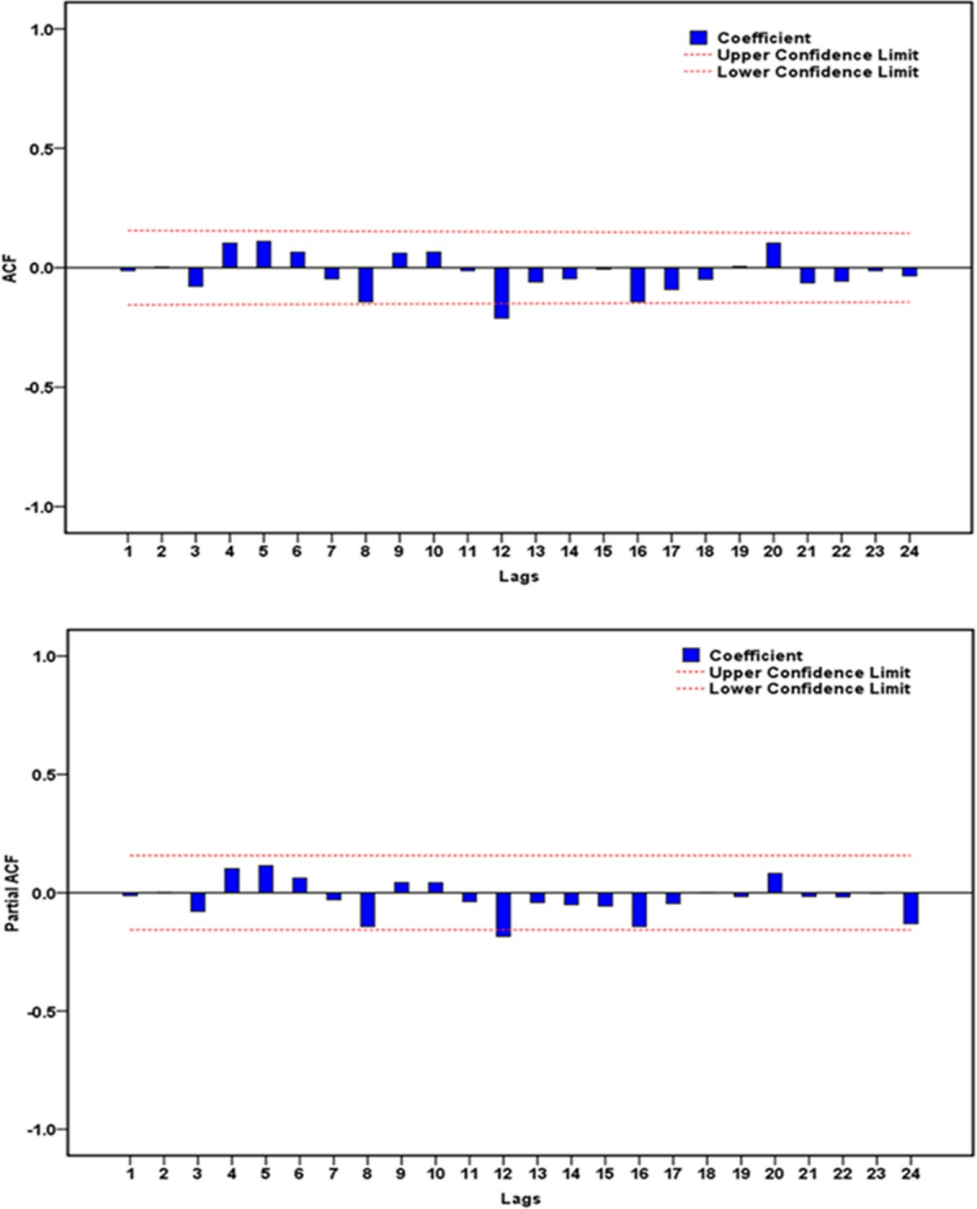
Autocorrelation function (ACF) and partial autocorrelation function (PACF) graphs of residuals from the hybrid model for the observatory pertussis morbidity time series. All spikes aside from the one at 12lag failed to exceed the estimated 95% uncertainty intervals, so the method appears to be suitable.

Here, in the case of satisfying the conditions for constructing the basic SARIMA and NAR models using the multi-scale components decomposed by the DWT. We attempted to evaluate the impact of the different mother wavelet types and decomposition levels on modeling accuracy. After simulating by trial and error, it can be seen from the data in **Tables 3 and 4**that the modeling results were satisfactory using the detailed and approximate sub-series obtained from the Daubechies wavelet(db2) at 1 and 2 levels as well as the Coiflets wavelet(coif1) at 1, 2 and 3 levels. Among the SARIMA models for each approximate subset showing the most outstanding fitting performance was the SARIMA(0,1,3)(2,0,1)_12_ specification at level 3 of coif1 wavelet(**Table 3**). While of all NAR methods for each detailed subset, the NAR model with 13 hidden neurons and 5 feedback delays was capable of enhancing the mimic accuracy at level 2 of coif1 wavelet(**Table 4**). However, when systematically taking into consideration the performances for different target series fitted and predicted by the wavelet based SARIMA-NAR combined model, the results, as displayed in **Table 5,** indicated that the hybrid approach at level 2 of db2 wavelet was superior to the others. Looking at **Fig 5**, it was apparent that the approximate element a2 stored the entire form of the chief signal, whereas the detailed components displayed most of the noise information of the reported pertussis series. The diagnostic results exhibited that the errors yielded by the corresponding individual models for the approximations and details belonged to a white noise(**Table 6, Fig 6 and S4 and S5 Figs**). In regard to the combined model, as can be seen from the **Table 1 and Fig 7,** the errors met the requirement of a stochastic white noise as well. Together these results confirmed that the developed combination model was worthy of being chosen as a rewarding tool to estimate the epidemic trends of pertussis in the coming year.

**Table 3:** The preferred SARIMA models’ parameters for various target series.

Target series	Approximation	SARIMA model	R^2^	Normalized BIC	Ljung-Box *Q*	
					Statistics	*P*
Original	-	SARIMA(2,1,0)(0,1,1)_12_	0.940	8.348	21.814	0.113
db2	a1	SARIMA(0,1,1)(0,1,1)_12_	0.976	7.362	22.307	0.134
db2	a2	SARIMA(0,1,3)(1,0,0)_12_	0.975	7.338	15.661	0.334
coif1	a1	SARIMA(0,1,2)(0,1,1)_12_	0.975	7.433	22.042	0.107
coif1	a2	SARIMA(1,1,4)(0,1,1)_12_	0.987	6.680	17.172	0.309
coif1	a3	SARIMA(0,1,3)(2,0,1)_12_	0.990	6.381	12.323	0.420

Original, pertussis incidence cases time series; db2, Daubechies wavelet; coif1, Coiflets wavelet; a1, a2 and a3, approximate components of the pertussis incidence cases time series generated by Daubechies or Coiflets wavelet; SARIMA, seasonal autoregressive integrated moving average model; R^2^, determination coefficient; BIC, Bayesian information criterion.

**Table 4:** The preferred NAR models’ parameters of various target series.

Target series	Levels	Hidden units	Delays	MSE			R			
				Training	Validation	Testing	Training	Validation	Testing	Overall
Original	-	16	6	3930.094	7460.696	5977.939	0.969	0.962	0.834	0.964
db2	1	16	5	104.066	404.740	96.919	0.953	0.953	0.936	0.947
db2	2(d1)	12	4	106.794	310.947	602.364	0.941	0.977	0.927	0.927
db2	2(d2)	11	5	41.833	218.312	1541.972	0.998	0.982	0.936	0.988
coif1	1	12	5	154.467	208.055	681.115	0.969	0.956	0.978	0.966
coif1	2(d1)	13	5	141.550	256.188	170.761	0.977	0.971	0.937	0.974
coif1	2(d2)	13	5	29.498	77.925	166.755	0.995	0.970	0.900	0.991
coif1	3(d1)	14	5	124.798	235.986	1627.252	0.970	0.954	0.946	0.954
coif1	3(d2)	12	5	56.408	266.313	199.657	0.990	0.958	0.938	0.982
coif1	3(d3)	15	5	341.436	932.159	279.393	0.984	0.953	0.992	0.982

Original, pertussis incidence cases time series; db2, Daubechies wavelet; coif1, Coiflets wavelet; d1, d2 and d3, detailed components of the pertussis incidence cases time series generated by Daubechies or Coiflets wavelet; MSE, mean square error; R, correlation coefficient.

**Table 5:** Performance assessment of various SARIMA-NAR hybrid models based on the DWT.

Mother wavelet type	Levels	Mimic part			Predicted part		
		MAPE	MAE	RMSE	MAPE	MAE	RMSE
db2	1	0.109	25.342	39.744	0.303	436.416	621.934
db2	2	0.085	22.854	38.097	0.067	76.006	92.015
coif1	1	0.135	29.659	41.363	0.493	538.029	617.032
coif1	2	0.080	19.571	28.081	0.219	345.398	621.438
coif1	3	0.094	23.682	34.031	0.189	243.336	348.098

DWT, discrete wavelet transform; db2, Daubechies wavelet; coif1, Coiflets wavelet; MAPE, mean absolute percentage error; MAE, mean absolute error; RMSE, root mean square error.

**Table 6:** Estimated parameters of the SARIMA(0,1,3)(1,0,0)_12_modelfor the approximation yielded by db2 wavelet.

Parameters	Coefficient	Standard error	*t*	*P*
MA1	-0.574	0.079	-7.240	<0.001
MA2	-0.282	0.091	-3.120	0.002
MA3	-0.339	0.081	-4.200	<0.001
SAR1	0.901	0.031	28.602	<0.001

MA1,moving average, lag1; MA2, moving average, lag2; MA3, moving average, lag3; SAR1, seasonal moving average, lag1.

### Comparison of mimic and predictive performances

**Fig 8:**
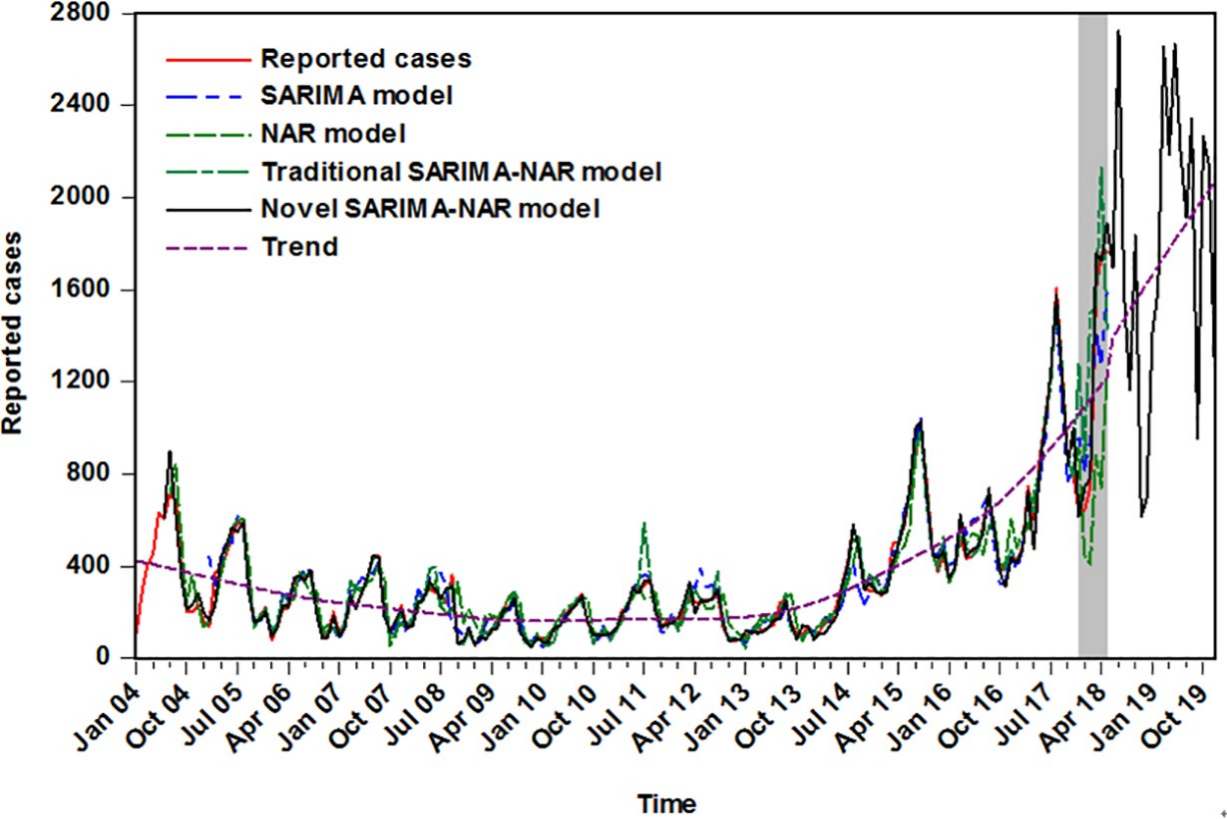
Comparison of incidence cases simulated and estimated between the selected four models and observations. The shaded area represents the validation data points from December 2017 to May 2018, in which the curve forecasted by the hybrid approach better tracked the actual; the black solid line stands for the trends from June 2018 to December 2019 projected by the hybrid method, there is a notably rising trend(purple dotted line).

The performance derived from the wavelet based SARIMA-NAR combined technique was compared with the single SARIMA, single NAR and traditional SARIMA-NAR methods from two facets of the simulation and forecasting. As illustrated in **Tables 7 and 8**, exhibiting that, in both fitting and predictive stages, our proposed hybrid model had the lowest error rates among these four models in terms of the MAPE, MAE, RMSE and MSE. In addition, **Fig 8** demonstrates that the curve simulated and forecasted by this data-driven hybrid approach was also the closest proximity to the actual. Therefore, the epidemic trajectories of pertussis from June 2018 to December 2019 were forecasted adopting this new method, being indicative of a seemingly continued upward trend of this disease.

**Table 7:** Predicting morbidity numbers of pertussis from December 2017 to May 2018 with the selected four models.

Time	Actual	SARIMA		NAR		Traditional SARIMA-NAR		Wavelet based SARIMA-NAR	
		Forecasted	AE	Forecasted	AE	Forecasted	AE	Forecasted	AE
December	627	957	0.459	915	0.459	1287	0.513	613	0.022
January	649	819	0.313	446	0.313	863	0.248	742	0.143
February	743	955	0.448	410	0.448	1503	0.506	783	0.054
March	1602	1427	0.451	880	0.451	1527	0.049	1757	0.097
April	1758	1267	0.577	743	0.577	2130	0.175	1726	0.018
May	1764	1589	0.296	1242	0.296	1431	0.233	1889	0.071

SARIMA, seasonal autoregressive integrated moving average model; NAR, nonlinear auto-regressive neural network model; Wavelet based SARIMA-NAR, integrating a seasonal autoregressive integrated moving model with a nonlinear autoregressive network model at level 2 of db2 wavelet; AE, absolute error.

**Table 8:** Comparison results of in-sample fitting and out-of-sample predicted performance using the selected four models.

**Models**	Mimic performance				Forecasted performance			
	MAPE	MAE	RMSE	MSE	MAPE	MAE	RMSE	MSE
SARIMA	0.178	43.169	61.245	3750.922	0.260	258.833	284.334	80845.833
NAR	0.234	47.362	66.966	4484.444	0.424	541.106	586.197	343627.309
Traditional SARIMA-NAR	0.122	26.075	40.190	1615.269	0.475	401.989	476.637	218684.000
Wavelet based SARIMA-NAR	0.085	22.854	38.097	1451.398	0.067	76.006	92.015	8466.756
**Reduced percentage (%)**								
Novel vs. SARIMA	52.247	47.059	37.796	61.306	74.231	70.635	67.638	89.527
Novel vs. NAR	63.675	51.746	43.110	67.635	84.198	85.954	84.303	97.536
Novel vs. Traditional	30.328	12.353	5.208	10.145	85.895	81.093	80.695	96.128

SARIMA, seasonal autoregressive integrated moving average model; NAR, nonlinear auto-regressive neural network model; Wavelet based SARIMA-NAR, integrating a seasonal autoregressive integrated moving model with a nonlinear autoregressive network model at level 2 of db2 wavelet; MAPE, mean absolute percentage error; MAE, mean absolute error; RMSE, root mean square error; MSE, mean square error.

## Discussion

With the emerging and reemerging foci occurrence of pertussis in China and worldwide over the past decade[10, 26]. This disease has brought about potential health hazards in newborns, together with potential losses to economy every year, and is being considered as a serious public health problem[27]. In order to better control and manage this life-threatening infection and achieve the ambitious goal of ending it. It is imperative to understand the temporal behaviors of the disease in advance, which will be of great importance to facilitate the allocation of medical and health resources. Little research has, however, concentrated on the utilization of a wavelet transform in the domain of epidemiological prediction. As a result, as far as we know, reported herein is the first study formulating a wavelet based SARIMA-NAR hybrid model to conduct forecasting in the incidence of pertussis. Meanwhile, the results emerged from our proposed combined approach were compared with three popular methods of the basic SARIMA, basic NAR and traditional SARIMA-NAR. The comparative results confirmed that the hybrid technique at level 2 of db2 wavelet has a notable advantage over, in both simulation and prediction subsets, the single SARIMA, single NAR and traditional SARIMA-NAR models in light of the minimum assessment indices including the MAPE, MAE, RMSE and MSE, which could enable these indices to slightly descend by 30.328%, 12.353%, 5.208% and 10.145%respectively in the fitting stage, and the counterparts in the forecasting stage to considerably plunge by 85.895%, 81.093%, 80.695% and 96.128% respectively compared to the traditional SARIMA-NAR approach. And by comparison with the basic SARIMA and NAR approaches, the above-mentioned indices also had a drastic reduction with regard to both the in-sample modeling points and out-of-sample predictive points. Likewise, the finding derived from the **Fig 8** exhibited that this hybrid model could better mirror the internal rules of pertussis epidemics as well. An earlier study observed that the Error-Trend-Seasonal(ETS) technique could also help to better improve predictions than that of a SARIMA method in the morbidity of pertussis[5]. When the wavelet based SARIMA-NAR technique was adopted to compare with the ETS approach used in the previous literature, in terms of the MAPE, MAE and RMSE, they still had a sharp reduction of 51.429%, 39.556% and 27.020% respectively in the facet of simulating, a further suggestion that our proposed combined approach can achieve a more accurate prediction for the morbidity of pertussis. Generally, as the data length and time periods used to develop models may have a significant influence on the forecasting accuracy, thus we further build this ETS model with our specified training and validation datasets. Similarly, be it in the training subset or in the validation subset, the wavelet based SARIMA-NAR technique can significantly better consider both the linear and non-linear clues to undertake forecasting for the seasonality and trend of pertussis(S2–S4 Tables). It is apparent that this data-driven combined method has a strong potential to boost the prediction of ability, since it allows us to seek the optimal combination method by decomposing a time-varying target series into different multi-scale levels to efficiently capture the complex linear and nonlinear characteristics in pertussis incidence. In this regard, our proposed wavelet based SARIMA-NAR hybrid model has provided a new insight into the epidemiological data prediction, it may offer the base data and theoretical support to build and assess the control measures of pertussis. The MAPE is commonly thought of as a key index to judge the accuracy of a forecast[28]: when the value is less than 10%, being suggestive of a highly accurate prediction. Exhilaratingly, such anticipated goal is also achieved by our proposed method. Meanwhile, considering the favorable performance and low-cost data gathering, this data-driven approach seems to be worthy of being extrapolated to detect and analyze the temporal trends of pertussis and even all types of infectious diseases in advance. However, it should be noted that with the rapid development of computational modeling techniques, numerous combined models have been emerged as a powerful tool in the domain of predictions, for instance, the hybrid techniques based on the ensemble empirical mode decomposition have widely been employed to forecast the annual runoff, land surface temperature and PM2.5 and the like[29]. Importantly, they have been proved to obtain the desired results. Hence, future comparative studies among these data-driven hybrid models will have to be conducted in order to approach the perfect prediction. In addition, in this work, we found that the traditional SARIMA-NAR approach outperformed the basic SARIMA and NAR methods in the simulating stage, whereas a contrary finding was observed in the forecasting stage. The present finding is not in good agreement with the past studies[13, 23], but the result from the present study agrees relatively well with that of a recent publication regarding the prediction of the number of new admission inpatients with the SARIMA-NAR technique[15].This finding means that the traditional combined technique does not necessarily have an advantage over its constituents’ performances, and this difference may be due to the data characteristics in different regions or time period. Meanwhile, contrary to our expectations, the basic NAR network significantly underperformed compared to the SARIMA method regarding the fitted and forecasted performances. These results further indicate that it is worthwhile exploring an appropriate method for different forms of data to attain traction as an early detection signal.

In the past decades, China has made great progress in preventing and controlling pertussis, and has been on track to eliminate this disease with target attainment recommended by the WHO, with a reduction in the mortality rate by 75% in 2015 relative to 2000[30]. In the present work, by characterizing monthly pertussis cases from January 2004 to May 2018 in China. Overall, it can be found that China has retained a low pertussis morbidity rate with yearly 0.277 per 100000 persons in the period of 2004 through 2017, this is much lower than some endemic countries[31]. But, since 2013, the pertussis case notifications have gradually commenced to skyrocket, especially during the period of January 2017 to May 2018, which has served as a wake-up call to deal with this urgent situation as quickly as possible for the Chinese public health agencies. Besides, whether a continuously rising trajectory in pertussis morbidity will be seen in the near future is still unknown. Therefore, the epidemic trends were detected adopting our identified best-performing combined method, indicating that the reported cases would be projected to continuously upsurge between June 2018 and December 2019. According to these findings, we can thus infer that China is afflicted with an increasingly chronic threat of pertussis. As for the manifest rebound in pertussis notifications, although the exact causes have not been completely elucidated. Several contributory factors may account for such a change. Firstly, the enhanced availability of diagnostic tests, like culture, PCR and serologic, has provided a deeper insight into the recognition of quite a few mildly or moderately affected subjects, particularly among adolescents and adults without typical symptoms[11, 32]. Secondly, the medical and public health professionals have reinforced awareness that the diagnosis and notification for the disease are vital as it is under mandatory to report the notifiable infectious diseases in time in China. Thirdly, there are various efficacies against pathogenic infection between whole cell and acellular vaccine, which may conduce to the adaptation or waning of pathogens to natural or vaccine immunity[33]. Evidence stemming from a slice of literature has proved that the provision of pertussis vaccine can function as precaution against causative agents with approximately 4–12 years, and the serum-specific antibody levels start to decline within 9 months exposed to antigen[8, 34]. Based on the vaccination procedure of pertussis in China[12], this situation exactly matches in temporality the fact that the infections in adolescents and adults have recently been in the growing notifications[10–12, 35, 36]. Fourthly, new pathogenic strains of pertussis with a chosen preponderance among the vaccinated subjects are now emerging[8]. Lastly, with the two-child policy officially implemented in China on January 1, 2016, the same year has witnessed a booming growth in newborns with about 1.31 million[37]. Furthermore, it is estimated that, due to this policy, the net increase in population will be more than 3 million and such an explosive growth will be continued until 2020[37]. Therefore, to discontinue the continued spread of pertussis, close attention should be paid to enhance the comprehensive interventions(e.g., strengthening the vaccine advocacy and education among adolescents, adults and pregnant women with low vaccine uptake) and further studies regarding the feasibility of some additional precautions will be imminently required(e.g. enhancing the vaccine efficacy and duration of prevention).

Seasonal distribution of diseases is among the most significant epidemiological features[38]. And pertussis has been recognized as a seasonal illness in many countries[38–43]. The results presented in our study demonstrated that the reported pertussis cases visibly varied by season with a periodicity. For example, a trough often occurred in January, February, October, November and December annually, among which there frequently existed the lowest incidence numbers in January, while the notified cases remained at a high level from March to September annually, with the highest level in August. Such a seasonal profile of pertussis notifications in China is also reported in other countries[39, 40, 43, 44]. Yet the results emerged from a retrospective analysis in the south and southeast regions of Brazil did not accord with our observations, which demonstrated that the months of peak and trough are contrary to our conclusions[42, 45]. Currently, the specific pathogenesis as to why the morbidity of pertussis varies by season still remains fully unclear. Plausible explanations may be that it is of great help in the mechanism of transmission and survival of the etiologic factor by offering an optimum environment and host, and the changes in epidemiology of the co-infected pathogens and organism immunologic function[38]. In addition, relevant literature has revealed that the opening of schools with the crowding of susceptible individuals may be responsible for such a seasonal variation in the morbidity of pertussis[40]. However, the relationship between this factor and the seasonality of pertussis needs to be further investigated in China.

The strengths shown in present study included that the conclusions are derived from the nationwide pertussis data from January 2004 to May 2018, and the verification of these data was supported by the mandatory reporting system of notifiable infectious diseases. However, still being subject to several weaknesses. Firstly, the notified cases of pertussis are confirmed by clinical or laboratory diagnoses. While the individuals with mild infection may frequently fail to go to medical institutions for diagnosis or treatment, thus resulting in a substantial under-reporting. Nevertheless, the obtained data have reflected the actual number of pertussis incidence to a great extent. Secondly, the demographic characteristics of the notified cases (e.g. age and sex) are missing, hence we are unable to obtain detailed information in data analysis. Thirdly, a variety of complicated driving factors even including some unpredictable drivers contribute to the occurrence of pertussis, whereas our models were established depended only on the retrospective response variable data without added explanatory variables due to their unavailability. Therefore, whether the models constructed with as many factors as possible have the potential to boost the mimic and predictive performances remain to be further authenticated. Fourthly, utilizing the reported time instead of the actual onset time may have a bearing on the seasonal changes of pertussis. Fifthly, the constructed model should be updated with the new cases in time. Finally, our proposed method was developed based on the nationwide case notifications. Therefore, the obtained results merely represented the country-level situation and trends of pertussis. After being documented, this new hybrid method is of great benefit to other regions or infectious diseases as well.

### Conclusions

To conclude, our proposed hybrid approach has gone distinctly some way towards enhancing the prediction accuracy, which can provide a valuable insight into the understanding of epidemic behaviors in pertussis incidence cases, and thus helping the health policymaker in rationally allocating health resources and appropriately developing the preventive and control planning for this disease. Furthermore, pertussis, as a seasonal illness in China, will be estimated to continue to rise and the case numbers are even high, this issue warrants to be urgently tackled strategically within the proper policy adopted.

## Supporting information

S1 Fig Systematic seasonal factors for pertussis notified cases at national level from January 2004 to May 2018 using the decomposition method. (TIF)

S2 Fig The regression plots of the basic NAR model’s outputs corresponding to targets for the training, validation, testing and whole datasets. (TIF)

S3 Fig The regression plots of the traditional SARIMA-NAR model’s outputs corresponding to targets for the training, validation, testing and whole datasets. (TIF)

S4 Fig The regression plots of the NAR model’s outputs corresponding to the detailed component d1 generated by db2 wavelet for the training, validation, testing and whole datasets. (TIF)

S5 Fig The regression plots of the NAR model’s outputs corresponding to the detailed component d2 generated by db2 wavelet for the training, validation, testing and whole datasets. (TIF)

S1 Table Time series of monthly pertussis notified cases in China from January 2004 to May 2018. (XLSX)

S2 Table Goodness of fit tests for different candidate models used to simulate the pertussis morbidity time series from January 2004 to November 2017. (DOCX)

S3 Table Initial parameters of the selected optimal ETS(A,N,A) model. (DOCX)

S4 Table Comparison results of in-sample fitting and out-of-sample predicted performance between wavelet based SARIMA-NAR model and ETS(A,N,A) model. (DOCX)
