# Supplementary figures and images for: Time series modeling of pertussis incidence in China from 2004 to 2018 with a novel wavelet based SARIMA-NAR hybrid model
Source: PLoS One. 2018 Dec 26;13(12):e0208404. doi: 10.1371/journal.pone.0208404 (PMC6306235; doi:10.1371/journal.pone.0208404)

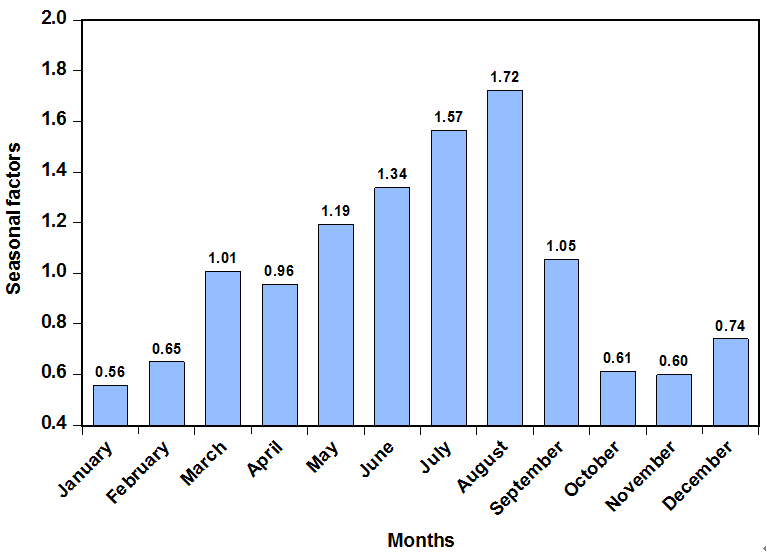

Supplement: S1 Fig — (TIF) [file pone.0208404.s001.tif]

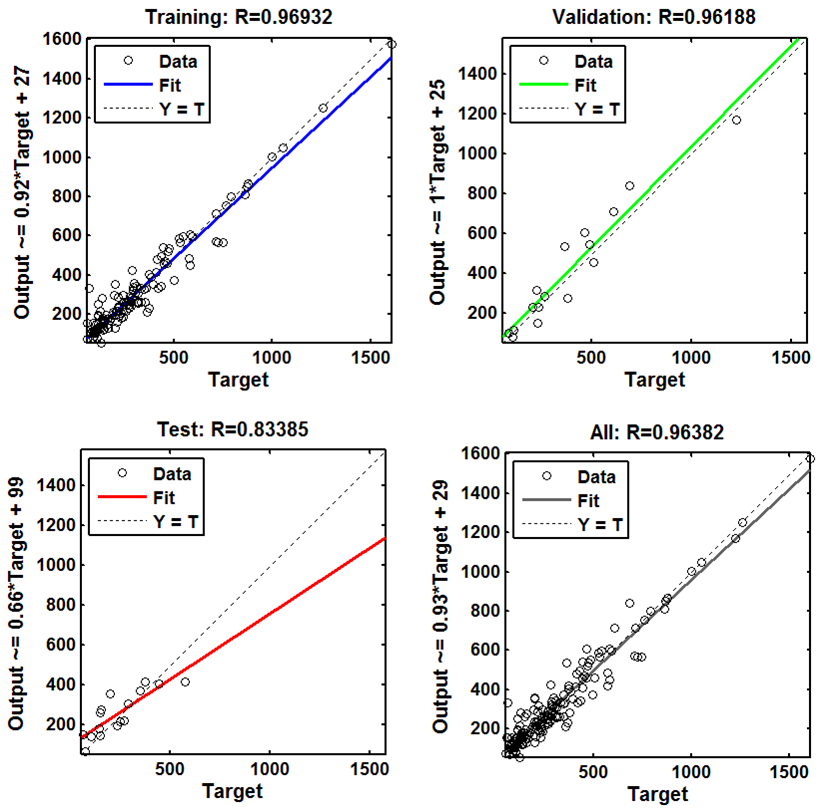

Supplement: S2 Fig — (TIF) [file pone.0208404.s002.tif]

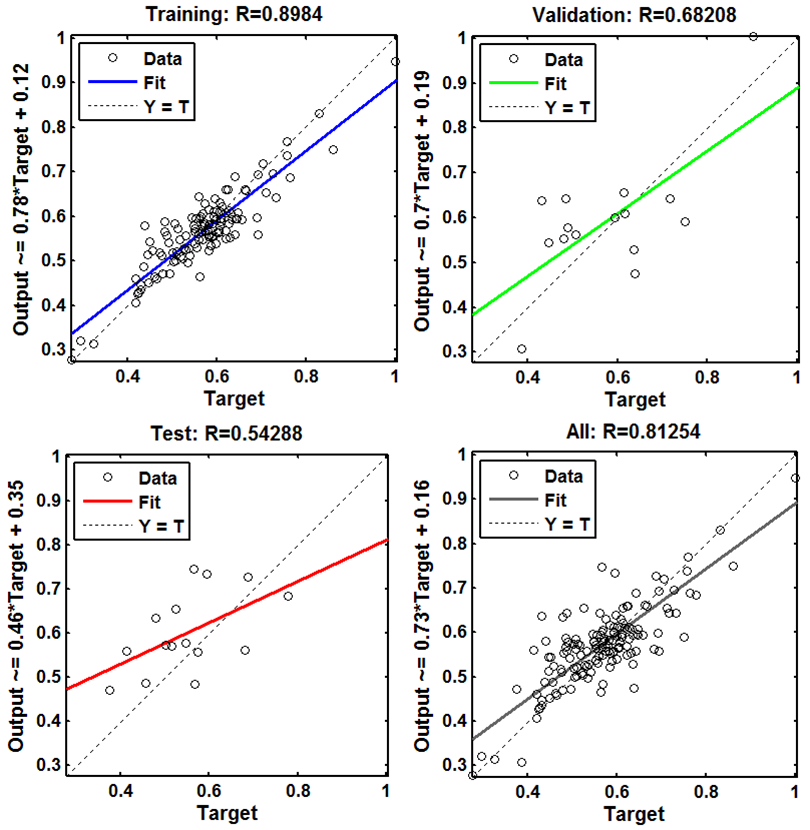

Supplement: S3 Fig — (TIF) [file pone.0208404.s003.tif]

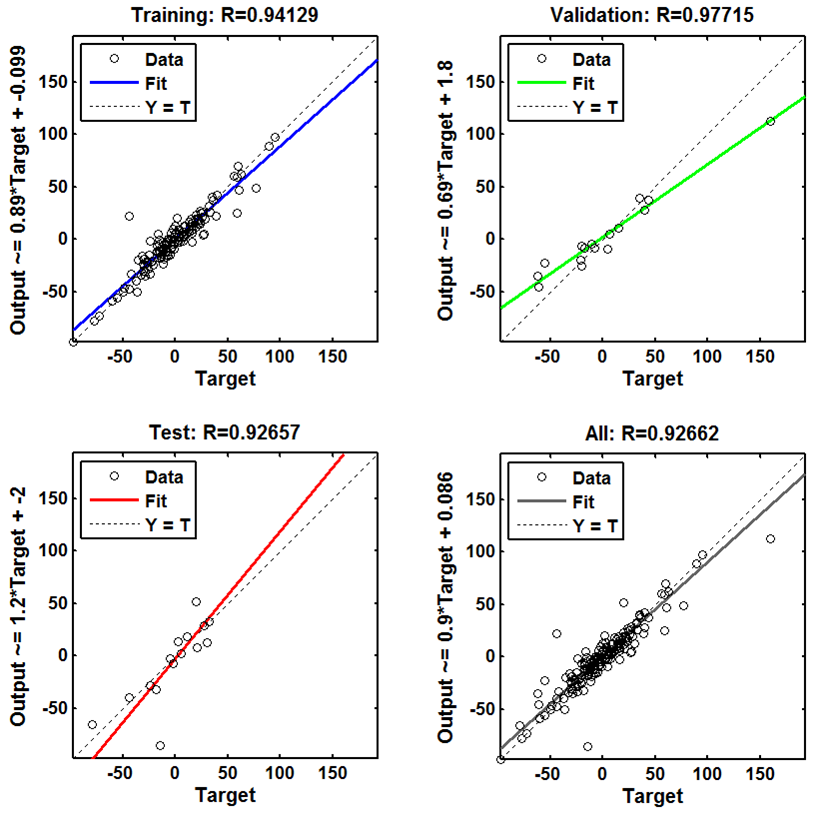

Supplement: S4 Fig — (TIF) [file pone.0208404.s004.tif]

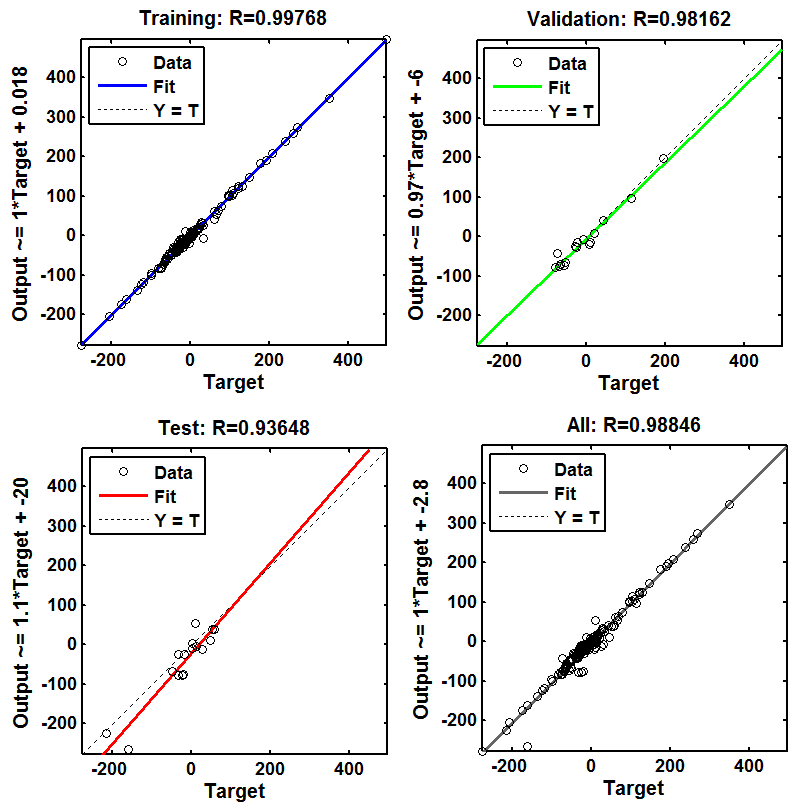

Supplement: S5 Fig — (TIF) [file pone.0208404.s005.tif]
