# Supplementary material for: Time series modeling of pertussis incidence in China from 2004 to 2018 with a novel wavelet based SARIMA-NAR hybrid model
Source: PLoS One. 2018 Dec 26;13(12):e0208404. doi: 10.1371/journal.pone.0208404 (PMC6306235; doi:10.1371/journal.pone.0208404)
Supplement: S2 Table — (DOCX) [file pone.0208404.s007.docx]

**S2 Table.** **Goodness of fit tests for different candidate models used to simulate the pertussis morbidity time series from January 2004 to November 2017.**

| **Models** | **Compact LL** | **Likelihood** | **AIC** | **BIC** | **HQ** | **AMSE** |
| --- | --- | --- | --- | --- | --- | --- |
| A,N,A | -1138.815 | -948.425 | 2305.629 | 2349.281 | 2323.347 | 10603.479 |
| A,AD,A | -1136.540 | -946.150 | 2307.079 | 2360.085 | 2328.593 | 1.E+100 |
| A,MD,A | -1136.616 | -946.226 | 2307.231 | 2360.237 | 2328.745 | 11167.291 |
| A,M,A | -1138.508 | -948.119 | 2309.017 | 2358.905 | 2329.265 | 10541.902 |
| A,A,A | -1138.550 | -948.161 | 2309.101 | 2358.989 | 2329.349 | 10504.795 |
| M,AD,N | -1159.217 | -968.827 | 2328.434 | 2344.024 | 2334.762 | 31605.442 |
| M,A,N | -1160.470 | -970.080 | 2328.940 | 2341.412 | 2334.002 | 33236.984 |
| M,MD,N | -1173.545 | -983.155 | 2357.090 | 2372.680 | 2363.418 | 47795.501 |
| M,M,N | -1178.361 | -987.972 | 2364.723 | 2377.195 | 2369.785 | 35303.966 |
| M,N,N | -1180.600 | -990.210 | 2365.200 | 2371.436 | 2367.731 | 29980.443 |
| M,N,A | -1187.333 | -996.943 | 2402.666 | 2446.318 | 2420.383 | 331978.713 |
| A,N,N | -1213.866 | -1023.477 | 2431.733 | 2437.969 | 2434.264 | 28011.924 |
| A,MD,N* | -1211.515 | -1021.126 | 2433.031 | 2448.620 | 2439.358 | 33336.338 |
| A,AD,N* | -1211.582 | -1021.192 | 2433.163 | 2448.753 | 2439.491 | 1.00E+100 |
| A,M,N | -1213.015 | -1022.625 | 2434.029 | 2446.501 | 2439.091 | 27290.297 |
| A,A,N* | -1213.752 | -1023.362 | 2435.503 | 2447.975 | 2440.565 | 27918.899 |
| A,MD,M | -1256.293 | -1065.903 | 2546.586 | 2599.592 | 2568.100 | 41645.725 |
| M,MD,A | -1265.086 | -1074.696 | 2564.172 | 2617.178 | 2585.686 | 26376.302 |
| M,M,A | -1267.648 | -1077.258 | 2567.296 | 2617.184 | 2587.545 | 62587.477 |
| M,MD,M | -1275.153 | -1084.764 | 2584.307 | 2637.313 | 2605.821 | 871275.406 |
| M,AD,A | -1290.405 | -1100.015 | 2614.810 | 2667.816 | 2636.324 | 1.00E+100 |
| M,A,A | -1298.199 | -1107.809 | 2628.398 | 2678.286 | 2648.646 | 30217.159 |
| A,AD,M* | -1490.568 | -1300.178 | 3015.135 | 3068.141 | 3036.649 | 1E+100 |
| M,N,M* | -1515.918 | -1325.528 | 3059.836 | 3103.488 | 3077.553 | 204992.400 |
| A,N,M* | -1518.630 | -1328.241 | 3065.261 | 3108.913 | 3082.978 | 257719.997 |
| A,A,M* | -1591.034 | -1400.645 | 3214.069 | 3263.957 | 3234.317 | 1260260.063 |
| M,A,M* | -1659.963 | -1469.573 | 3351.925 | 3401.813 | 3372.174 | 34481068.089 |
| M,AD,M* | -1732.027 | -1541.637 | 3498.054 | 3551.059 | 3519.567 | 1.00E+100 |
| M,M,M* | -2932.089 | -2741.699 | 5896.178 | 5946.066 | 5916.427 | 5.11E+39 |
| A,M,M* | -3274.616 | -3084.226 | 6581.231 | 6631.119 | 6601.479 | 7.71816E+15 |

***Note:*** *11 models failed to converge; Compact LL, Compact Log-likelihood; AIC, Akaike Information Criterion; BIC, Schwarz Criterion; HQ, Hannan-Quinn Criterion; AMSE, Average Mean Squared Error. Selection among ETS processes can be done by the performance indices including the Compact LL, AIC, BIC, HQ and AMSE. The best-fitting ETS(A,N,A) model based on the comprehensive consideration with the smallest AIC, BIC, HQ and AMSE and the largest Compact LL and Likelihood this chosen. Besides, the detail descriptions regarding the ETS model can be found in the reference [[1](#_ENREF_1)].

1. Hyndman RJ, Khandakar Y. Automatic Time Series Forecasting: The forecast Package for R. Journal of Statistical Software. 2008;27(3):1-22.
