# Supplementary material for: Time series modeling of pertussis incidence in China from 2004 to 2018 with a novel wavelet based SARIMA-NAR hybrid model
Source: PLoS One. 2018 Dec 26;13(12):e0208404. doi: 10.1371/journal.pone.0208404 (PMC6306235; doi:10.1371/journal.pone.0208404)
Supplement: S3 Table — (DOCX) [file pone.0208404.s008.docx]

**S3 Table. Initial parameters of the selected optimal ETS(A,N,A) model.**

| **Parameters** | **Values** |
| --- | --- |
| Alpha: | 1 |
| Gamma: | 0 |
| **Initial Parameters** | |
| Initial level: | 247.853 |
| Initial state 1: | -81.990 |
| Initial state 2: | -119.435 |
| Initial state 3: | -109.062 |
| Initial state 4: | 42.025 |
| Initial state 5: | 220.683 |
| Initial state 6: | 162.770 |
| Initial state 7: | 96.571 |
| Initial state 8: | 54.657 |
| Initial state 9: | -19.256 |
| Initial state 10: | -0.098 |
| Initial state 11: | -105.011 |
| Initial state 12: | -141.852 |
| **Performance** | |
| Compact Log-likelihood | -1138.815 |
| Log-likelihood | -948.425 |
| Akaike Information Criterion | 2305.629 |
| Schwarz Criterion | 2349.281 |
| Hannan-Quinn Criterion | 2323.347 |
| Sum of Squared Residuals | 837769.800 |
| Root Mean Squared Error | 70.828 |
| Average Mean Squared Error | 10603.479 |
