# Supplementary material for: Time series modeling of pertussis incidence in China from 2004 to 2018 with a novel wavelet based SARIMA-NAR hybrid model
Source: PLoS One. 2018 Dec 26;13(12):e0208404. doi: 10.1371/journal.pone.0208404 (PMC6306235; doi:10.1371/journal.pone.0208404)
Supplement: S4 Table — (DOCX) [file pone.0208404.s009.docx]

**S4 Table. Comparison results of in-sample fitting and out-of-sample predicted performance between wavelet based SARIMA-NAR** **model and ETS(A,N,A) model.**

| **Models** | **Mimic performance** | | | |  | **Forecasted performance** | | | |
| --- | --- | --- | --- | --- | --- | --- | --- | --- | --- |
|  | **MAPE** | **MAE** | **RMSE** | **MSE** |  | **MAPE** | **MAE** | **RMSE** | **MSE** |
| ETS(A,N,A) | 0.218 | 50.383 | 70.828 | 5016.586 |  | 0.328 | 456.807 | 566.687 | 321134.608 |
| Wavelet based SARIMA-NAR | 0.085 | 22.854 | 38.097 | 1451.398 |  | 0.067 | 76.006 | 92.015 | 8466.756 |
| **Reduced percentage (%)** | | | | | | | | | |
| Novel vs. ETS | 61.009 | 54.639 | 46.212 | 71.068 |  | 79.573 | 83.361 | 83.763 | 97.363 |

Wavelet based SARIMA-NAR, integrating a seasonal autoregressive integrated moving model with a nonlinear autoregressive network model at level 2 of db2 wavelet; ETS, Error-Trend-Seasonal technique; MAPE, mean absolute percentage error; MAE, mean absolute error; RMSE, root mean square error; MSE, mean square error.
